# Parabens and Human Epidermal Growth Factor Receptor Ligand Cross-Talk in Breast Cancer Cells

**DOI:** 10.1289/ehp.1409200

**Published:** 2015-10-27

**Authors:** Shawn Pan, Chaoshen Yuan, Abderrahmane Tagmount, Ruthann A. Rudel, Janet M. Ackerman, Paul Yaswen, Chris D. Vulpe, Dale C. Leitman

**Affiliations:** 1 Department of Nutritional Sciences and Toxicology, University of California, Berkeley, Berkeley, California, USA; 2 Silent Spring Institute, Newton, Massachusetts, USA; 3 Life Sciences Division, Lawrence Berkeley National Laboratory, Berkeley, California, USA

## Abstract

**Background:**

Xenoestrogens are synthetic compounds that mimic endogenous estrogens by binding to and activating estrogen receptors. Exposure to estrogens and to some xenoestrogens has been associated with cell proliferation and an increased risk of breast cancer. Despite evidence of estrogenicity, parabens are among the most widely used xenoestrogens in cosmetics and personal-care products and are generally considered safe. However, previous cell-based studies with parabens do not take into account the signaling cross-talk between estrogen receptor α (ERα) and the human epidermal growth factor receptor (HER) family.

**Objectives:**

We investigated the hypothesis that the potency of parabens can be increased with HER ligands, such as heregulin (HRG).

**Methods:**

The effects of HER ligands on paraben activation of c-Myc expression and cell proliferation were determined by real-time polymerase chain reaction, Western blots, flow cytometry, and chromatin immunoprecipitation assays in ERα- and HER2-positive human BT-474 breast cancer cells.

**Results:**

Butylparaben (BP) and HRG produced a synergistic increase in c-Myc mRNA and protein levels in BT-474 cells. Estrogen receptor antagonists blocked the synergistic increase in c-Myc protein levels. The combination of BP and HRG also stimulated proliferation of BT-474 cells compared with the effects of BP alone. HRG decreased the dose required for BP-mediated stimulation of c-Myc mRNA expression and cell proliferation. HRG caused the phosphorylation of serine 167 in ERα. BP and HRG produced a synergistic increase in ERα recruitment to the c-Myc gene.

**Conclusion:**

Our results show that HER ligands enhanced the potency of BP to stimulate oncogene expression and breast cancer cell proliferation in vitro via ERα, suggesting that parabens might be active at exposure levels not previously considered toxicologically relevant from studies testing their effects in isolation.

**Citation:**

Pan S, Yuan C, Tagmount A, Rudel RA, Ackerman JM, Yaswen P, Vulpe CD, Leitman DC. 2016. Parabens and human epidermal growth factor receptor ligand cross-talk in breast cancer cells. Environ Health Perspect 124:563–569; http://dx.doi.org/10.1289/ehp.1409200

## Introduction

Xenoestrogens are a class of synthetic estrogens known as endocrine-disrupting chemicals that bind to estrogen receptors in cells to mimic or antagonize the action of endogenous estrogens, such as 17β-estradiol (E2) (Zoeller et al. 2012). Numerous xenoestrogens are found in common household products, including plastics, food and soda cans, and personal-care products. One class of xenoestrogens that is of increasing public health concern is esters of parahydroxybenzoic acid, commonly known as parabens (Nohynek et al. 2013; Karpuzoglu et al. 2013). These compounds are common ingredients in cosmetics, shampoos, body lotions, and sunscreens, where they are used to prevent microbial growth and prolong shelf life (Dodson et al. 2012; Guo and Kannan 2013). Detectable levels of multiple parabens are present in human urine (Calafat et al. 2010; Den Hond et al. 2013; Mortensen et al. 2014) and breast tissue (Barr et al. 2012; Darbre et al. 2004; Darbre and Harvey 2014).

Endocrine-disrupting chemicals have been linked to a variety of medical conditions; one of the most troubling is their association with breast cancer (Darbre and Harvey 2008; Vandenberg et al. 2012; Zoeller et al. 2012). Endogenous estrogens promote breast cancer by binding to estrogen receptor α (ERα) (Burns and Korach 2012; Sommer and Fuqua 2001), which causes the activation of oncogenes, such as *c-Myc* and *cyclin D1* (Leygue et al. 1995; Liao and Dickson 2000). Cyclin D1 and c-Myc cause cell proliferation by facilitating a G1 to S-phase transition (Foster et al. 2001). Approximately two-thirds of breast tumors express ERα, and therapeutic strategies aimed at preventing and treating ER-positive breast tumors are directed at blocking the action of ERα. Parabens are known to bind to ERα (Routledge et al. 1998), promote a G_1_ to S-phase cell cycle progression, stimulate the proliferation of MCF-7 breast cancer cells (Darbre et al. 2003; Okubo et al. 2001; Wróbel and Gregoraszczuk 2013), and activate transcription of cell cycle (Wróbel and Gregoraszczuk 2014) and reporter genes (Darbre et al. 2003; Gomez et al. 2005). These findings indicate that paraben exposure might increase the risk of breast cancer by activating ERα to promote the activation of proliferative genes. However, parabens are considered to be safe because of their weak estrogenic binding affinity, transcriptional activation, and stimulation of cell proliferation. Furthermore, the dose required for ERα activation often exceeds the amount found in the body (Lemini et al. 2003; Pugazhendhi et al. 2005). The most estrogenic paraben, butylparaben, was found to be 10,000-fold less potent than E2 (Routledge et al. 1998). However, studies involving xenoestrogens have tested them in the absence of activators of the human epidermal growth factor receptor (HER) family of receptor tyrosine kinases (Wróbel and Gregoraszczuk 2013, 2014), a second signaling pathway implicated in breast cancer (Liu et al. 2009).

The HER family comprises four receptors: EGFR/HER1, ErbB2/HER2, ErbB3/HER3, and ErbB4/HER4 (Davoli et al. 2010). HER2 is a transmembrane protein that is overexpressed in ~25% of breast tumors (Davoli et al. 2010). Its presence in human tumors is a negative prognostic indicator because it is associated with malignant transformation, fast growth, and more aggressive tumors (Barros et al. 2010; Davoli et al. 2010). The association between HER2 expression and breast cancer led to the development of the drug Herceptin® (trastuzumab), a recombinant humanized monoclonal antibody against HER2, to treat HER2-positive tumors (Hudis 2007). At least 11 proteins, known as HER ligands, can bind to HER family members to cause dimerization, leading to the activation of the phosphoinositide 3-kinase/protein kinase B (PI3K/AKT) pathway and other signal transduction pathways (Mosesson and Yarden 2004). Aberrant activation of the PI3K/AKT signaling pathway may increase the risk of cancer by inhibiting apoptosis and stimulating cell proliferation (Liu et al. 2009). HER and ERα signaling pathways can cross-talk, as indicated by the observation that HER ligands stimulate phosphorylation of the serine 167 (Ser167) residue in ERα (Al-Dhaheri and Rowan 2006; Lannigan 2003; Murphy et al. 2011). Eliminating the main source of endogenous estrogens by ovariectomy delays the formation of mammary tumors and increases the lifespan of transgenic mice that overexpress HER2 in the mammary gland (Anisimov et al. 2003). Furthermore, when HER2 transgenic mice are mated to ERα knockout mice, tumor onset is delayed compared with control HER2 transgenic mice (Hewitt et al. 2002). Based on these findings, we hypothesized that activators of the HER2 pathway might cause parabens to stimulate ERα at lower doses than suspected given the results of studies that examined their effects in isolation. In the present study, we determined the potency of parabens in the presence of the HER ligand HRG in BT-474 breast cancer cells that express both ERα and HER2.

## Materials and Methods

### Cell Culture

Human BT-474, MCF-7, and SKBR3 breast cancer cell lines were obtained from ATCC and were used in these studies because of differences in their expression of HER2 and ERα. BT-474 cells are HER2-negative and ERα-positive, MCF-7 cells are ERα-positive and HER2-negative, and SKBR3 cells are HER2-positive and ERα-negative (Neve et al. 2006). Cells were grown in phenol red–free Dulbecco’s modiﬁed Eagle’s medium/F12 (DMEM/F12) supplemented with 10% fetal bovine serum (FBS), 2 mM L-glutamine, 100 U/mL penicillin, and 10 μg/mL streptomycin (Life Technologies) under 5% CO_2_ at 37°C. Three days before treatment, the cells were incubated with DMEM/F12 supplemented with 10% charcoal-dextran–stripped FBS (Gemini Bio-Products). Recombinant human heregulin-β1 (HRG) was purchased from Leinco Technologies, Inc. and used at a final concentration of 20 ng/mL to activate the HER2 signaling pathway. Estradiol, raloxifene, tamoxifen, methylparaben (MP), ethylparaben (EP), propylparaben (PP), and butylparaben (BP) were purchased from Sigma-Aldrich Co. LLC. The estrogen receptor antagonist ICI 182,780 was purchased from Tocris Bioscience. The compounds were dissolved in ethanol. The final concentration of ethanol was 0.1%, which had no effect on the cells. An ethanol vehicle was used for the control cells.

### Real-Time RT-PCR

BT-474 cells (passage numbers 86–95) were grown in six-well tissue culture dishes to reach 80% confluence and then maintained in DMEM/F12 supplemented with 10% charcoal-dextran–stripped FBS for 3 days. The cells were treated with 0.01 μM E2 or 10 μM MP, EP, PP or BP in the absence or presence of 20 ng/mL HRG for 2 hr. The 10 μM concentration of parabens was selected by performing preliminary dose–response studies. Total RNA was isolated and purified using an Aurum Total RNA Mini Kit (Bio-Rad Laboratories Inc.). RNA purity and concentration were determined using a NanoDrop ND-1000 spectrophotometer (Thermo Fisher Scientific Inc.). Reverse transcription of total RNA was carried out using iScript (Bio-Rad Laboratories Inc.) as previously described (Paruthiyil et al. 2009). SsoFast EvaGreen Supermix (Bio-Rad Laboratories Inc.) was used for PCR and DNA amplification of the *c-Myc* and glyceraldehyde 3-phosphate dehydrogenase (*GAPDH*) genes with a Bio-Rad CFX96 Real-Time System. The following PCR primers were used:

*GAPDH* Forward 5´-CGA​TGC​TGG​CGC​TGA​GTA​CG​T-3´; *GAPDH* Reverse 5´-CCT​GCA​AAT​GAC​CCC​CAG​CCT​TC-3´; *c-Myc* Forward 5´-GGA​AAA​CCA​GCA​GCC​TCC​CGC​-3´; *c-Myc* Reverse 5´-ACG​GCT​GCA​CCG​AGT​CGT​AG-3´. The expression of *c-Myc* and *GAPDH* was determined by the comparative Ct method as previously described (Paruthiyil et al. 2009).

### Western Blot

Human BT-474, MCF-7, and SKBR3 cells were grown in six-well tissue culture dishes in phenol red–free DMEM/F12 supplemented with 10% FBS, 2 mM L-glutamine, 100 U/mL penicillin, and 10 μg/mL streptomycin under 5% CO_2_ at 37°C. Three days before treatment (80% confluence), the medium was replaced with DMEM/F12 supplemented with 10% charcoal-dextran–stripped FBS. The cells were then treated for 2 hr with increasing concentrations of BP in the absence and presence of 20 ng/mL HRG. Cells were lysed in radioimmunoprecipitation assay (RIPA) buffer containing 50 mM Tris-HCl (pH 7.4), 150 mM NaCl, 1% NP-40; 0.5% sodium deoxycholate; 0.1% sodium dodecyl sulfate (SDS), and cOmplete™ Protease Inhibitor Cocktail (Roche Diagnostics). The total protein concentration was determined with the Coomassie Plus™ Protein Assay Reagent (Thermo Fisher Scientific Inc.). Fifteen micrograms of cell proteins from each sample were then separated by SDS–polyacrylamide gel electrophoresis (SDS-PAGE) and transferred to a polyvinylidene difluoride (PVDF) membrane. The membrane was blocked with 5% nonfat dry milk in Tris-buffered saline with Tween 20 (TBST) [20 mM Tris-HCl (pH 7.5), 500 mM NaCl, and 0.1% Tween 20] and probed overnight with rabbit anti-c-Myc IgG (sc-764; Santa Cruz Biotechnology Inc.) at 0.5 μg/mL in 1% milk-TBST at 4°C. After washing with TBST for 5 min for three times at 22°C, the membrane was incubated with goat anti-rabbit IgG conjugated to horseradish peroxidase (sc-2054; Santa Cruz Biotechnology, Inc.) at 1:10,000 dilution in 1% milk-TBST for 1 hr at room temperature. Proteins were visualized using the Amersham ECL Prime Western Blotting Detection Reagent (GE Healthcare Life Sciences). The Western blot for ERα phosphorylation was performed as described for c-Myc except that the cell lysis buffer contained a phosphatase inhibitor cocktail (PhosSTOP; Roche Diagnostics) and the primary antibody was anti-phospho-ERα S167 (Bethyl Laboratories).

### Chromatin Immunoprecipitation (ChIP)

Confluent BT-474 cells were treated with butylparaben in the absence and presence of HRG (20 ng/mL) for 1–3 hr. The cells were harvested for a ChIP assay as previously described (Cvoro et al. 2006) with some modifications. Briefly, to cross-link proteins to DNA, the cells were fixed by adding formaldehyde to the cell culture medium and were then incubated at 37°C for 10 min; then, the cross-linking reaction was quenched by the addition of glycine for 5 min at room temperature. The cell monolayer was then washed with phosphate-buffered saline (PBS) containing cOmplete™ Protease Inhibitor Cocktail and collected by scraping. The cells were concentrated by centrifugation and lysed as previously described by Vivar et al. (2010) with buffer containing 0.5% Triton X-100, 50 mM Tris-HCl (pH 7.4), 150 mM NaCl, 10 mM EDTA, and cOmplete™ Protease Inhibitor Cocktail Tablets. The cell lysate was centrifuged at 2000 × *g* for 5 min, and the pellets were resuspended in RIPA buffer containing cOmplete™ Protease Inhibitor Cocktail Tablets. Each sample was sonicated on ice to shear genomic DNA, and the samples were incubated with 4 μg/mL rabbit anti-ERα IgG (sc-544; Santa Cruz Biotechnology) or the same concentration of normal rabbit IgG (sc-2025; Santa Cruz Biotechnology) at 4°C overnight with rotation. Immunoprecipitation was performed with Protein G Mag Sepharose (GE Healthcare) according to the manufacturer’s instructions. The protein-DNA complex was eluted in 1% SDS and 0.1 M NaHCO_3_, and cross-linking of protein-bound DNA was reversed by incubating the samples at 65°C overnight. DNA was purified using a ChIP DNA Clean & Concentrator kit (Zymo Research). ERα antibody–precipitated DNA was amplified by real-time PCR with specific primers for the *c-Myc* enhancer region as previously described (Wang et al. 2011).

### Cell Cycle Analysis using Flow Cytometry

The effects of different treatments on cell cycle phase were analyzed by flow cytometry based on a previously described method (Wiepz et al. 2006). Briefly, BT-474 cells were plated at 500,000 cells per well in six-well tissue culture dishes with phenol red–free DMEM/F12 supplemented with 10% stripped FBS. Forty-eight hours later, the culture medium was switched to serum-free DMEM/F12. After 24 hr of synchronization with serum-free DMEM/F12, the cells were treated with the indicated concentrations of BP (see figure legends) in the presence and absence of HRG (20 ng/mL) for 24 hr. The cells were then trypsinized and collected by centrifugation at 300 × *g* for 5 min at room temperature. The cell pellets were washed once with ice-cold PBS and centrifuged at 1,700 rpm for 10 min at room temperature followed by resuspension in 500 μL of propidium iodide solution (PBS containing 0.1% Triton 100, 0.1% sodium citrate, 10 μg/mL RNase, and 0.05 mg/mL propidium iodide) to stain the cells. The cell suspensions were assayed using a Cytomics FC-500 flow cytometer with CXP software (Beckman Coulter, Inc.) in the flow cytometry core facility at University of California, Berkeley, and the data were then analyzed using FlowJo 7.6.5 (FlowJo, Inc.).

### Cell Proliferation Assay

BT-474 cells were plated in six-well tissue culture dishes in phenol red–free DMEM/F12 supplemented with 10% charcoal-dextran–stripped FBS. The next day, the cells were treated with increasing concentrations (0.01–1 μM) of BP in the absence or presence of HRG (20 ng/mL) and incubated for 1 or 5 days without changing the medium. After treatment, the cells were washed with PBS and detached from the plates with trypsin and then placed in ISOTON® diluent and counted with a Coulter Counter (Beckman Coulter, Inc.).

### Statistical Analysis

Data are presented as the mean ± SD or the mean ± SEM as indicated in the figure legends. The statistical significance of the differences was determined by one- or two-way analysis of variance (ANOVA) as specified in the figure legends. All ANOVA tests were followed by *post hoc* Tukey’s multiple comparison tests to analyze the differences between time periods or doses within groups treated with the same reagents (BP, HRG, or BP plus HRG). Bonferroni’s multiple comparison *post hoc* test was applied to analyze the differences between groups with and without HRG within the same paraben treatment group or within the same time period. Data analysis was performed using GraphPad Prism (v.6.01; GraphPad Software, Inc.).

## Results

### Combined Effects of Parabens and HRG on c-Myc *Transcript Levels in BT-474 Breast Cancer Cells*

**Figure 1:**
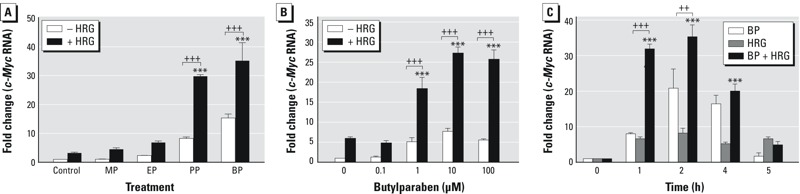
Effects of parabens and heregulin (HRG) on *c-Myc* transcript levels in BT-474 breast cancer cells. (*A*) Human BT-474 breast cancer cells were treated with 10 μM methylparaben (MP), 10 μM ethylparaben (EP), 10 μM propylparaben (PP), or 10 μM butylparaben (BP) for 2 hr in the absence and presence of HRG (20 ng/mL). (*B*) BT-474 breast cancer cells were treated with 0.01 μM 17β-estradiol (E2) or increasing concentrations (0.1 μM to 100 μM) of BP in the absence and presence of HRG (20 ng/mL) for 2 hr. (*C*) BT-474 breast cancer cells were treated with 10 μM BP in the absence or presence of HRG (20 ng/mL) for the indicated time periods. Relative mRNA levels for *c-Myc* were determined by real-time polymerase chain reaction (PCR) and were normalized to *GAPDH* using the comparative CT method. The fold changes were obtained by comparing the treated values with the control values. Each data point is the average of triplicate samples ± SEM. The figures are representative of three experiments with similar results. The statistical significance of the means was analyzed by two-way analysis of variance (ANOVA) followed by a *post hoc *Tukey’s multiple comparisons test to analyze the significance of the differences between the control and various parabens (*A*), various doses of BP (*B*), and various time periods (*C*) in the presence of HRG. Differences in *c-Myc *expression comparing cells receiving the same paraben treatment (*A*), the same dose of BP (*B*), and the same time period (*C*), but with and without HRG, were tested using a *post hoc *Bonferroni multiple comparisons test. ***p < 0.001. Bonferroni multiple comparisons test: ^++^ *p *< 0.01 and ^+++^ *p *< 0.001.

Because BT-474 cells express both ERα and HER2 (Lazaro et al. 2013), they represent a suitable cell model to explore the interactions between ERα and HER2 signaling pathways. BT-474 cells were treated with MP, EP, PP, and BP, all of which are commonly present in cosmetics and in personal-care products, in the absence and presence of HRG. Of the parabens listed above, PP and BP were the most effective at increasing *c-Myc* mRNA expression in the absence of HRG (Figure 1A). HRG alone produced an approximately 3-fold increase in *c-Myc* mRNA expression, but a synergistic increase that was greater than additive was observed with PP and BP (Figure 1A). BP was the most effective stimulator of *c-Myc* mRNA expression in the absence and presence of HRG and was selected for further studies. The maximal increase of *c-Myc* expression by BP was observed at a concentration of 10 μM (Figure 1B). The synergistic effect of HRG was observed when BT-474 cells were treated with BP for 1 hr (Figure 1C). These results indicate that HRG decreased the dose required for the BP-mediated increase in *c-Myc* mRNA expression and enhanced the magnitude of the BP response.

### Combined Effects of HRG and BP on c-Myc Protein Levels in ERα Positive Cell Lines

**Figure 2:**
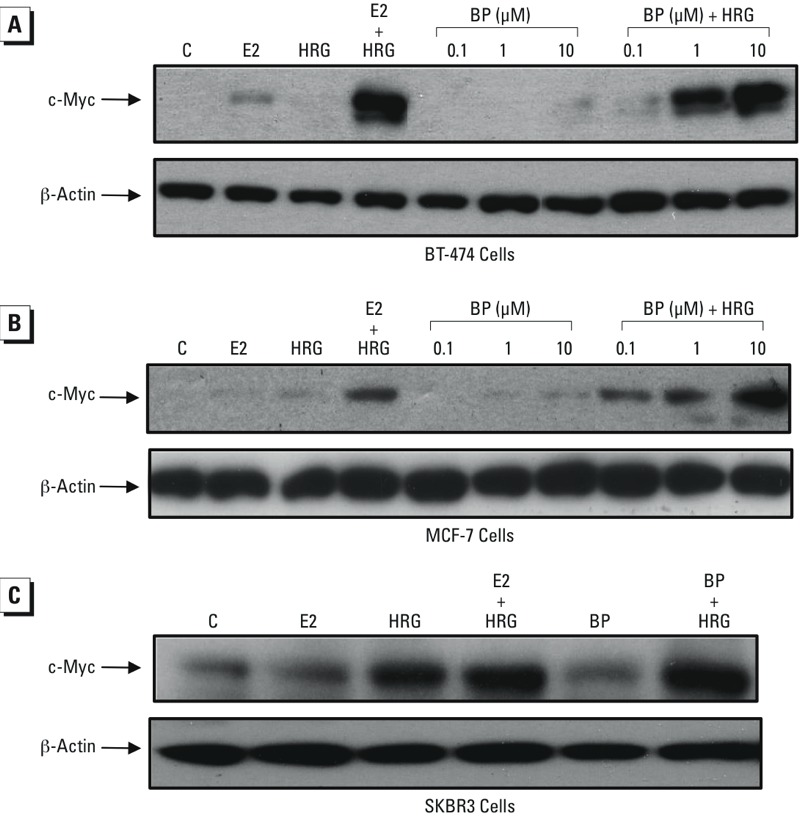
Effects of heregulin (HRG) on butylparaben (BP) stimulation of c-Myc protein levels in breast cancer cell lines. (*A*) BT-474 and (*B*) MCF-7 breast cancer cells were treated with the indicated concentrations of BP without or with HRG (20 ng/mL) for 2 hr. (*C*) SKBR3 cells were treated with 10 μM BP in the absence and presence of HRG (20 ng/mL) for 2 hr. The treated cells were then lysed, and the cellular lysates were prepared for Western blots using antibodies against c-Myc as described in “Materials and Methods.” Cells simultaneously treated with 17β-estradiol (E_2_) (0.01 μM) were also included for comparison. After exposure to an X-ray film, the membranes were washed and reprobed with an antibody against β-actin as a loading control. This figure is representative of two independent experiments with similar results.

**Figure 3:**
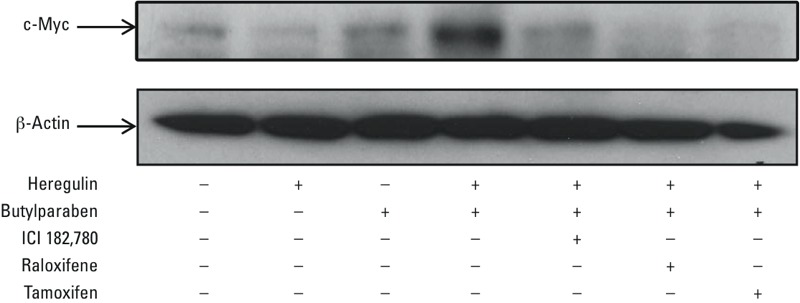
Effects of estrogen receptor antagonists on the synergistic increase of c-Myc protein levels. BT-474 cells were treated with 10 μM butylparaben (BP) in the absence or presence of 1 μM ICI 182,780, 1 μM raloxifene, 10 μM tamoxifen, and 20 ng/mL HRG for 2 hr. The treated cells were then lysed, and the cellular lysates were prepared for Western blots using antibodies against c-Myc as described in “Materials and Methods.” After exposure to an X-ray film, the membranes were washed and reprobed by an antibody against β-actin as the loading control. This figure is representative of two independent experiments with similar results.

To determine the effects of HRG on BP stimulation of c-Myc protein production, BT-474 cells were treated with HRG in the absence or presence of increasing concentrations of BP for 2 hr. No increase in c-Myc protein levels was observed with BP or HRG (Figure 2A) alone. In the presence of HRG with 1 μM and 10 μM BP, the increase in c-Myc protein levels was similar to that induced by 0.01 μM E2 plus HRG. Similarly to the BT-474 cells, in MCF-7 cells, which express ERα but not HER2, enhanced BP induction of c-Myc protein levels was observed with HRG (Figure 2B). In contrast, in the SKBR3 cell line, which is HER2-positive and ERα-negative, no synergistic increase in c-Myc protein levels was observed. The increase in c-Myc protein levels that occurred in BT-474 cells with BP and HRG was blocked by the estrogen receptor antagonists ICI 182,780, raloxifene, and tamoxifen (Figure 3). These results indicate that HRG potentiates BP stimulation of c-Myc only in ERα-positive breast cancer cells and that the potentiation requires ERα signaling.

### Combined Effects of HRG and BP on BT-474 Cell Proliferation

**Figure 4:**
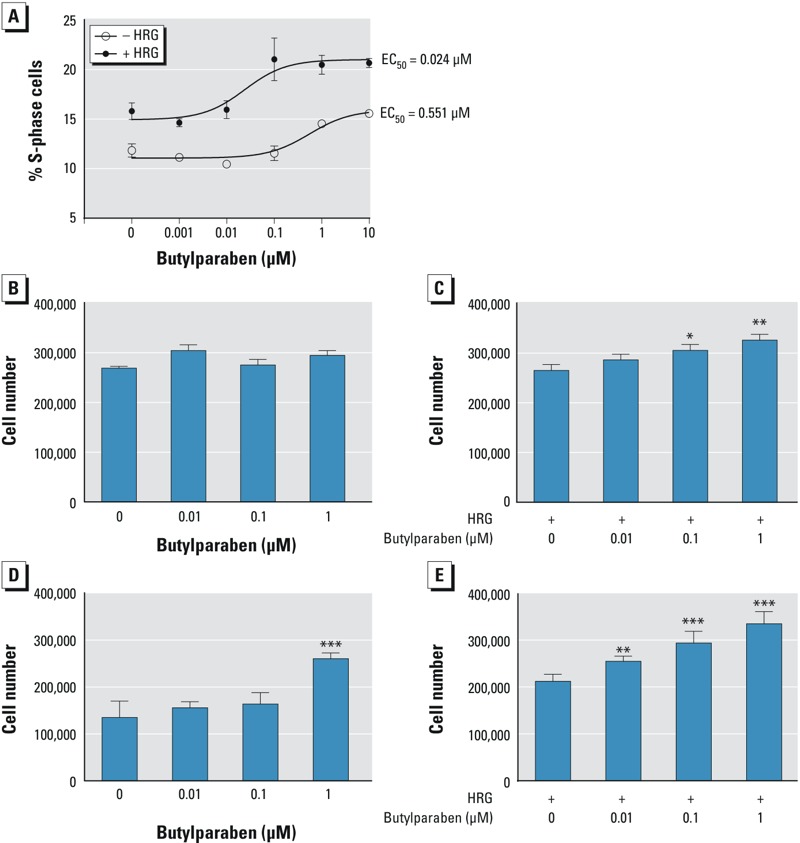
Effects of heregulin (HRG) on the potency of butylparaben (BP) stimulation of BT-474 cell proliferation. (*A*) BT-474 cells were treated with the indicated concentrations of BP alone or BP and HRG for 24 hr. Changes in cell cycle distribution were then analyzed by flow cytometry as described in “Materials and Methods.” The percentage of S-phase cells was plotted for different BP concentrations in the absence and presence of HRG. The plotted values are the means (± SD) of biological triplicates, which represent three independent experiments with similar results. (*B*,*C*) BT-474 cells were plated in 6-well dishes at 250,000 cells/well. The culture medium was then switched to serum-free medium 24 hr later, and the cells were treated with the indicated concentrations of BP in the absence (*B*) or presence (*C*) of HRG (20 ng/mL). The cells were harvested after 24 hr of treatment and then counted with a Coulter counter. The figures are representative of three experiments with similar results. The data are expressed as the means (± SD) obtained from biological triplicates. Statistical significance was analyzed by one-way analysis of variance (ANOVA) followed by a *post hoc *Tukey’s multiple comparison test to compare the differences between the control and each dose. (*C*) *p < 0.05 and **p < 0.01 compared with HRG alone. (*D*,*E*) BT-474 cells were plated in 6-well dishes at 50,000 cells/well and treated with indicated amounts of BP in the absence (*D*) or presence (*E*) of HRG. The cells were harvested after 5 days of treatment and then counted with a Coulter counter. The data are expressed as means (± SD) obtained from biological triplicates and are representative of three experiments with similar results. The statistical significance was analyzed with one-way ANOVA followed by a *post hoc *Tukey’s multiple comparison test to compare the differences between the control and each dose of BP with or without HRG. (*D*) ***p < 0.001 compared with the untreated control. (*E*) **p < 0.01 and ***p < 0.001 compared with cells treated with HRG alone.

The effects of HRG on BP stimulation of BT-474 cell proliferation were examined using flow cytometry. BT-474 cells were treated with BP alone or with HRG plus BP for 24 hr. DNA content in the cells was measured by flow cytometry. Treatment with BP alone at a concentration of 1 μM increased the number of cells entering S-phase (Figure 4A). The addition of HRG resulted in increased potency of BP. The EC_50_ for BP alone was 0.551 μM, whereas the EC_50_ for BP plus HRG was 0.024 μM (Figure 4A). To compare the results obtained from the flow cytometry study, we counted the cells with a Coulter counter after 24 hr treatment with BP in the absence and presence of HRG. BP alone did not stimulate cell proliferation after 24 hr (Figure 4B). A significant increase in cell number occurred with 0.1 and 1 μM BP in the presence of HRG (Figure 4C). The shift in BP potency was more pronounced after treatment for 5 days. In the absence of HRG, 1 μM BP was required to produce a significant increase in cell number (Figure 4D), whereas in the presence of HRG, 0.01 μM BP significantly increased cell number (Figure 4E). These findings indicate that HRG lowered the dose of BP required to stimulate BT-474 cell proliferation.

### Effects of HRG on Serine 167 Phosphorylation of ERα and the Recruitment of ERα to the c-Myc *Enhancer by HRG plus BP*

**Figure 5:**
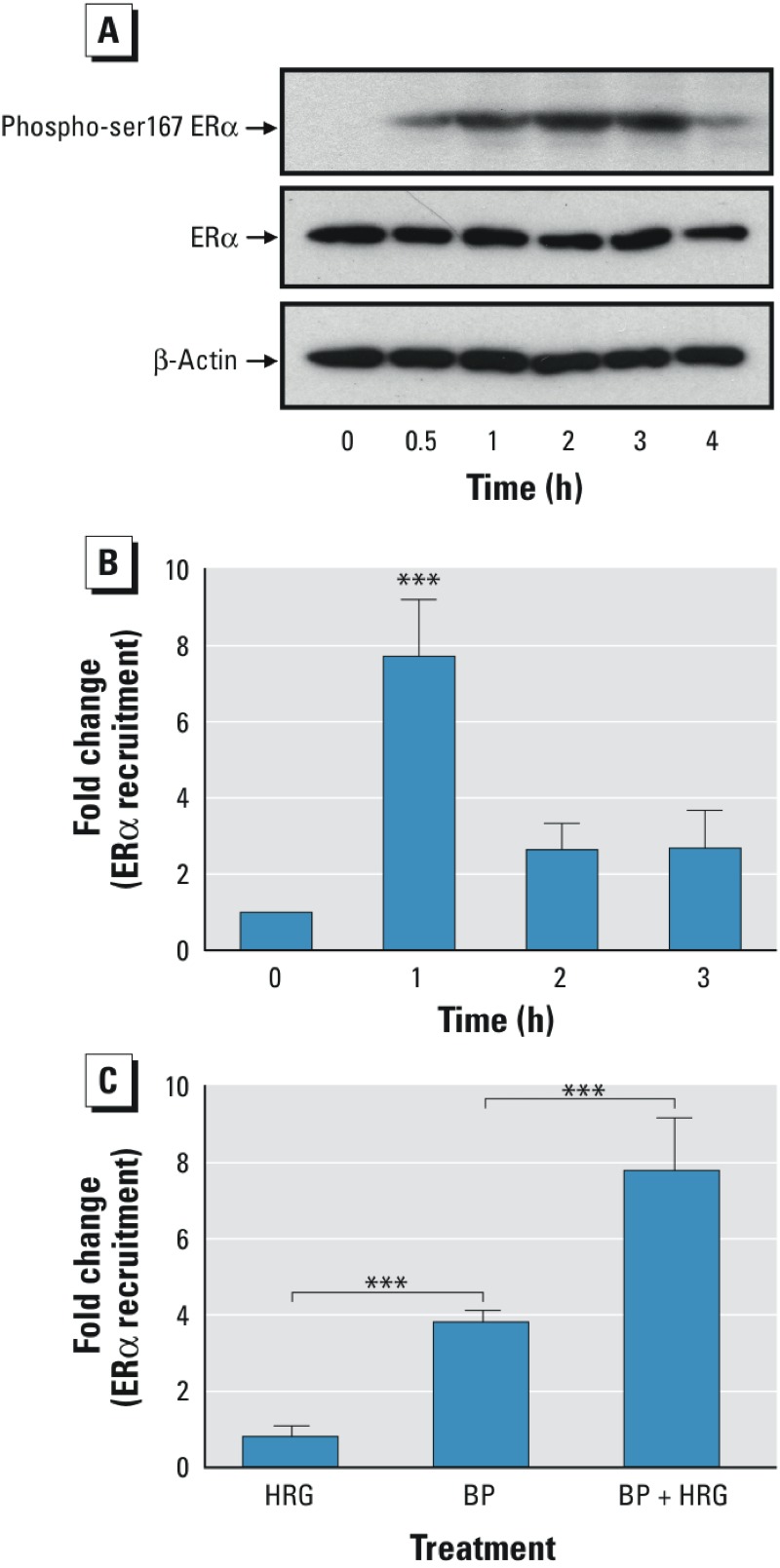
Effects of heregulin (HRG) on the phosphorylation of serine 167 (Ser167) in estrogen receptor α (ERα) and the recruitment of ERα to the *c-Myc* enhancer. (*A*) BT-474 cells were treated with HRG (20 ng/mL) for 0.5, 1, 2, 3, and 4 hr. The treated cells were lysed and subjected to Western blotting using an anti-ERα Ser167 phosphorylation antibody to probe phosphorylated ERα. Total ERα expression in the treated cells was also determined by Western blotting with anti-ERα monoclonal antibody. (*B*) BT-474 cells were treated with BP (10 μM) in the presence of HRG (20 ng/mL) for 1, 2, and 3 hr. The ERα recruitment to the *c-Myc* enhancer was examined by performing ChIP assays with anti-ERα antibody. (*C*) BT-474 cells were treated with HRG (20 ng/mL), butylparaben (BP) (10 μM), or BP plus HRG for 1 hr and were then subjected to ChIP assays with anti-ERα antibody. The data (*B,C*) shown are derived from quantitative real-time polymerase chain reaction (PCR) analysis of an ERα binding site in the *c-Myc* enhancer region. The Ct values of ERα antibody–precipitated DNA were adjusted by nonspecific immunoprecipitated DNA. The fold changes were obtained by comparing the adjusted Ct values of treated samples with those of a nontreated sample (control). The results are expressed as the mean ± SD from triplicate experiments. The statistical significance was analyzed with one-way analysis of variance (ANOVA) followed by a *post* *hoc *Tukey’s multiple comparison test to compare the differences between the control at each time point (*B*) and between the control and HRG, BP, and HRG plus BP. ***p < 0.001.

One potential mechanism whereby HRG and BP could cooperate to produce a synergistic activation of *c-Myc* expression is through phosphorylation of ERα and subsequent enhanced binding of ERα to the *c-Myc* gene. To explore this possibility, the phosphorylation of serine 167 (Ser167) in ERα was assessed by Western blotting of BT-474 cells treated with HRG for increasing lengths of time. HRG caused a detectable increase in the phosphorylation of Ser167 in ERα at 30 min, and a maximal response was obtained at 2 hr (Figure 5A). HRG did not change the level of the unphosphorylated form of ERα or the levels of β-actin. The recruitment of ERα to a known ER binding site in the *c-Myc* enhancer element was examined by ChIP in BT-474 cells after treatment with HRG and BP. A maximal 8-fold enhancement of ERα binding to the *c-Myc* enhancer sequence was observed after 1 hr (Figure 5B). The increase in ERα binding was greater in cells treated with both HRG and BP than in cells treated with HRG or BP alone (Figure 5C). These results show that the combination of HRG and BP increased both ERα phosphorylation and the recruitment of ERα to the *c-Myc* enhancer.

## Discussion

HER2 and ERα are components of two major signaling pathways that are often altered in breast cancers (Nair et al. 2012). Xenoestrogens can mimic endogenous estrogens to promote the proliferation of breast cancer cells (Jenkins et al. 2012). Most studies have investigated the effects of xenoestrogens alone on end points such as cell proliferation (Wróbel and Gregoraszczuk 2013, 2014). However, the biological effects of xenoestrogens, particularly at low doses, might be altered in the presence of factors that activate other signaling pathways that can cross-talk with estrogen receptors (Schiff et al. 2004). For example, growth factors such as HRG and EGF activate downstream Akt signaling, which causes the phosphorylation of Ser167 in ERα (Joel et al. 1998; Nagashima et al. 2008). Phosphorylation of ERα plays a critical role in gene transcription by enhancing ligand binding to ER, nuclear localization, dimerization, DNA binding, and coactivator recruitment (Al-Dhaheri and Rowan 2006; Lannigan 2003; Murphy et al. 2011). Based on these findings, we hypothesized that studies using parabens and other xenoestrogens alone could underestimate their proliferative effects in breast tissue cells and their potency to promote breast cancer, particularly at lower doses.

A major rationale promulgated in favor of the safety of xenoestrogens in consumer products is that at biologically relevant concentrations, they bind to estrogen receptors with too low an affinity to produce significant biological effects in humans (Golden et al. 2005). For example, BP was found to bind to ERα with ~10,000-fold lower affinity than E2 (Bolger et al. 1998). Similarly, functional assays of ERα such as reporter assay activation and MCF-7 cell proliferation found that physiologically implausible concentrations of parabens are needed for ERα activation (Golden et al. 2005). However, this argument does not take into account the possibility that other signaling pathways in cells, particularly those that promote cell proliferation, might potentiate paraben and other xenoestrogen effects by sensitizing ERα to activation at lower doses. In the present study, we investigated whether parabens are more potent in the presence of HER ligands. We demonstrated that HRG and BP could produce a synergistic increase in mRNA expression by the oncogene *c-Myc*. The increase in *c-Myc* mRNA expression was accompanied by a corresponding increase in c-Myc protein levels. The synergy required the presence of ERα because the synergy was blocked by estrogen receptor antagonists, and no synergy was observed in the ERα-negative, HER2-positive SKBR3 cell line. Unexpectedly, we observed that synergistic activation occurred in HER2-negative MCF-7 cells; this finding suggests that other receptors from the HER family can mediate the effects of HRG on estrogenic sensitivity, as discussed below. An HRG-mediated increase in the potency of BP was observed in two different proliferative assays. Our results revealed that HRG lowered the dose of BP required to significantly affect proliferation of ER-positive breast cancer cells.

Although it is clear that endogenous estrogens increase the risk of breast cancer, the role of parabens in breast cancer is controversial, in part because of uncertainty about whether the concentrations of parabens that are present in the body are sufficient to mimic the effects of endogenous estrogens on breast tissue cells (Harvey 2003; Karpuzoglu et al. 2013; vom Saal et al. 2007). In this study, we demonstrated that even in the presence of HRG, higher concentrations of parabens than those of E2 were needed to stimulate *c-Myc* expression and to cause proliferation of BT-474 cells. However, the PP and BP concentrations at which we observed estrogenic effects in the presence of HRG are within the range of concentrations previously reported in human breast tissue (Barr et al. 2012). Large-scale biomonitoring studies have reported urinary paraben concentrations ranging from 0.001 to 1 μM (Calafat et al. 2010; Frederiksen et al. 2011), although the relationship between urine and tissue levels remains uncertain. Furthermore, breast tissues may contain multiple parabens (Darbre et al. 2004), and combinations of different parabens can produce additive effects on proliferation (Charles and Darbre 2013). We found that HRG acted synergistically with PP and BP to increase *c-Myc* gene expression. Further studies will be needed to determine the effects of HRG in the presence of combinations of parabens or other xenoestrogens.

The presence of multiple HER receptors and ligands in breast tissues may affect the activity of parabens. The 11 known endogenous HER ligands can bind to one or more of the HER receptors (Mosesson and Yarden 2004) with the notable exception of HER2, for which there is no known ligand (Harari and Yarden 2000). However, the binding of ligands to HER1, HER3, or HER4 leads to a preferential dimerization and activation of HER2 (Rubin and Yarden 2001). Further work will be needed to determine if other HER ligands potentiate the effects of parabens and to determine their relative potency compared with that of HRG. Interestingly, breast cancer cells are autocrine producers of HER ligands. In a study of 363 breast tumors, it was found that 80%–96% of the tumors expressed at least one of ten tested HER ligands (Révillion et al. 2008). Similarly, another study found that 48% of breast tumors express HRG (Esteva et al. 2001). Breast tumors may therefore potentiate their own response to estrogenic compounds by producing HER ligands.

## Conclusion

Our data showing that lower doses of BP were required to stimulate breast cancer cell proliferation in the presence of HRG together with the observations that breast tumors are exposed *in vivo* to both HER ligands (Révillion et al. 2008) and parabens (Darbre et al. 2004) indicate a potential synergy relevant to the proliferation of tumor cells in humans. Further work is needed to assess whether HER ligands indeed enhance the potency of parabens in normal human breast tissue cells and in breast tumors. In light of our findings, we suggest that reevaluation of the potency of other xenoestrogens in the presence of HER ligands is warranted.
